# A comprehensive guide to research protocols for collecting and coding involuntary past and future thoughts

**DOI:** 10.1016/j.mex.2024.102732

**Published:** 2024-04-23

**Authors:** Krystian Barzykowski, Ewa Ilczuk, Lia Kvavilashvili

**Affiliations:** a Applied Memory Research Laboratory, Institute of Psychology, Faculty of Philosophy, Jagiellonian University, Kraków, Poland; b University of Hertfordshire, Hatfield, UK

**Keywords:** Vigilance task, Involuntary thoughts, Thought probes, Mind wandering, Spontaneous cognition, Involuntary autobiographical memories, Involuntary future thoughts, The Involuntary Thought Program

## Abstract

The paper presents a comprehensive guide for researchers investigating mind-wandering and related phenomena such as involuntary past and future thinking. Examining such spontaneous cognitions presents a challenge requiring not only the use of appropriate laboratory-based procedures, but also the coding of complex qualitative data. This guide outlines two main stages of existing research protocols: data acquisition and data coding. For the former, we introduce an easily modifiable computerized version of the vigilance task, designed for broad application in studies focusing on eliciting and measuring involuntary thoughts in controlled laboratory conditions. Regarding data preparation and coding, we provide a detailed step-by-step procedure for categorizing and coding different types of thoughts, involving both participants and competent judges. Additionally, we address some of the difficulties that may arise during this categorization and coding process. The guide is supplemented by a clip demonstrating the main part of the experimental procedure and a step-by-step example of the subsequent data processing stages. We anticipate that this research guide will not only assist a broader group of researchers interested in investigating spontaneous cognition, but will also inspire future studies on spontaneous cognition and related phenomena.

• There is a need for standardized approaches to working with qualitative data when investigating spontaneous thoughts.
• The paper outlines a comprehensive protocol for collecting and coding involuntary past and future-oriented thoughts.
• The paper also presents a detailed step-by-step procedure for data preparation and coding to categorize different types of thoughts, involving both participants and competent judges.

## Graphical abstract

A graphical abstract was submitted as a separate file. Note accompanying the graphical abstract: The graphical abstract presented is conceptual in nature and serves as an illustrative example. It does not include verbatim wording of the procedure. For further details and comprehensive information, please refer to the paper and accompanying materials.

**Figure:**
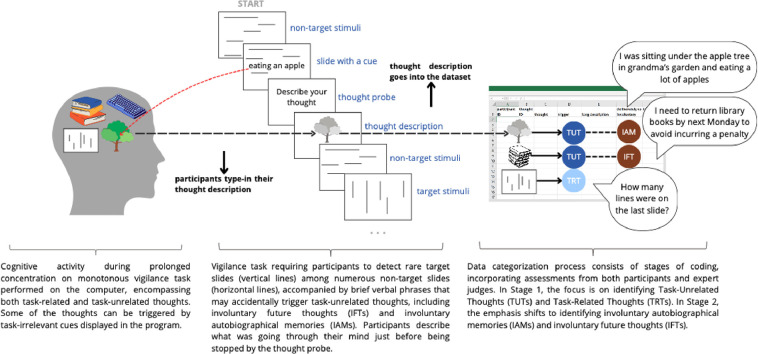

Specifications Table

**Table:** 

Subject area:	Psychology
More specific subject area:	Spontaneous thoughts, spontaneous cognition, involuntary past and future thoughts, mind wandering
Name of your method:	The Involuntary Thought Program
Name and reference of original method:	Barzykowski, K., Ilczuk, E., & Kvavilashvili, L. (2022). The role of working memory in the occurrence of involuntary thoughts about the past and future: An experimental investigation. Journal of Experimental Psychology: Learning, Memory and Cognition. [Registered Report Accepted In-Principle: Stage 1].
Resource availability:	N.A.

## Method details

 

## Introduction

Involuntary thoughts are spontaneous mental representations that come to one's mind without deliberate intention to retrieve them. They encompass a diverse range of mental contents and are frequently prompted by incidental cues in one's environment or internal stream of thought (e.g., [[1], [2], [3]]), especially when people are not engaged in attentionally demanding activities (e.g., [4,5]). The exploration of spontaneous thoughts encompasses various research fields, including investigations into involuntary autobiographical memories (e.g., [[6], [7], [8], [9]]), spontaneous future thinking (e.g., [10]), mind wandering (e.g., [11,12]) and rumination (e.g., [13,14]).

Developing a standardized experimental procedure that captures inherently spontaneous processes poses a significant challenge. Consequently, in recent years researchers have discussed methodological issues that arise when empirically studying and measuring involuntary thoughts [[15], [16], [17], [18]]. Several different methods have been used to study such thoughts and memories including the diary method, where respondents report their thoughts as they occur during everyday activities (so-called self-caught method) (e.g., [19]), questionnaire and survey methods (Bernsten & Rubin [20,21]), and experimental laboratory-based methods such as the Sustained Attention to Response Task (SART) that has been used extensively in research on mind-wandering. In this method, participants have to respond to digits from 1 to 9, but withhold their response to a particular digit (e.g., 3) and, while being engaged in this task, they are stopped and have to report and categorise the nature of their thoughts – specifically, whether they were related or unrelated to the task – without, however, disclosing the content of the thoughts (e.g., [22]). A different laboratory procedure was developed by Schlagman and Kvavilashvili [17] to study involuntary past and future thoughts. In their design, participants are engaged in a minimally demanding vigilance task while reporting their thoughts either every time they occur during the vigilance task (referred to as the self-caught method; e.g., [17]) or are prompted to write down the content of their thoughts at predetermined fixed time intervals (referred to as the probe-caught method; [23]).

The fundamental principle guiding laboratory-based research for studying involuntary thoughts is that participants are not trying to deliberately retrieve them, as this would compromise the involuntary nature of their occurrence. To ensure involuntary retrieval that is not contaminated by deliberate retrieval attempts, it is necessary to engage participants in cognitively undemanding tasks and collect involuntary thoughts that accompany the task performance. Various methods may be employed to engage participants in task performance, all with the shared objective of collecting the thoughts that participants experience during the laboratory procedure. This not only poses a challenge in utilising an appropriate laboratory-based procedure, but also presents researchers with complex qualitative data that require further processing and analysis.

The procedure, outlined in the present protocol, features a fully computerized and easily adaptable version of a laboratory paradigm developed for the study of involuntary past and future thoughts, but is applicable to research on mind-wandering in general. Although researchers may opt for various procedures, the data processing remains consistent across these methodologies, especially when they involve handling participants' entries describing the contents of their thoughts. Hence, in the present protocol, we specifically focus, for the first time in the literature, to a highly important but neglected aspect of research on spontaneous cognitions: the processing of the collected data. This procedure and data coding protocol have already been employed in several studies [[6], [16], [24], [25], [26], [27]], and its current version is a result of extensive work over several years. In our view, any research involving the analysis of spontaneous thoughts, particularly those with a quantitative focus, will benefit considerably from employing such a protocol. Its high level of automation, flexibility and potential for application make it a valuable resource. We are confident that offering this research guide will not only help a wider group of researchers, interested in investigating spontaneous cognition in general, but will also inspire future studies on spontaneous cognition and related phenomena.

## General description of the study and the procedure

The present protocol stems from a study exploring the relationship between working memory load and the frequency of involuntary autobiographical memories (IAMs) and involuntary future thoughts (IFTs) [28]. In this study, participants’ working memory load (none, low, high) was manipulated while they were completing a letter version of the N-back task during the undemanding vigilance task. The frequency of involuntary past and future-oriented thoughts was assessed by random thought-probes. We examined the hypothesis that the occurrence of IAMs and IFTs decreases with an increase in working memory load. Here, we primarily concentrate on the standard version of the procedure completed by participants in the control condition (i.e., only the vigilance task with random thought-probes). The modifications of the standard procedure used in experimental conditions, which involved additional processing of N-back task stimuli, will be briefly described in the section on possible modifications.

A total of 240 young adults, aged between 18 and 35 years, took part in the study in exchange for a modest financial reward (PLN 50, approximately $13). During the recruitment process, participants were not informed that the study investigated spontaneous thoughts about the past and future. The study was advertised as the "study investigating the focus of attention" to ensure that we collected reliable data that was unaffected by participants intentionally retrieving these thoughts during the ongoing task completion [[9], [16]]. Following recruitment, participants were randomly assigned to one of three conditions: a control group with no working memory load and two experimental groups with low and high working memory loads, respectively. The study was conducted in a controlled laboratory setting to minimise the effects of external distractions and confounding variables. Participants were tested in groups of two to twelve people in a single laboratory-based session. Participants followed the procedure on individual computer stations. It is noteworthy that, in our study, preparing computer keyboards involved affixing red squares on the "m" key in accordance with the instructions. While not mandatory, this procedure simplifies participants' responses by ensuring they press the correct button during the task. The entire experimental session lasted approximately 2 h, with the vigilance task segment of the main computer procedure taking an average of 1 hour and 15 min. Participants received a brief introduction to the procedure from the experimenter and then read detailed instructions displayed on the computer screen.

The primary component of the study involved a computerized vigilance task procedure. The program was developed using the Unity Real-Time Development Platform. Briefly, participants were tasked with identifying 15 infrequent target slides featuring vertical lines among a larger set of non-target slides displaying patterns of horizontal lines (785 slides in total). While engaged in the task, participants were exposed to 270 short verbal phrases, some of which could incidentally trigger task-unrelated thoughts, including IFTs and IAMs. During the vigilance task, participants were probed 23 times at random intervals to capture their thoughts at the moment of interruption and indicate whether the recorded thought had occurred spontaneously or deliberately. The visual presentation of components of the vigilance task are shown in Fig. 2 and video file #1. Upon completing the vigilance task, participants were provided with the descriptions of their thoughts, one by one, and indicated whether these thoughts referred to past memories or future events. Upon the study's completion, the collected thoughts underwent several stages of coding, involving expert judges, to ultimately identify IAMs and IFTs. The process of preparing data and categorization will be elaborated on in detail later in the paper.

## Computerized version of vigilance task

In this section, we will provide a detailed presentation of the procedure. As mentioned above, there are three key elements in the procedure:

1. the presence of a low-demand ongoing task (the vigilance task involves detecting rarely occurring vertical lines among non-target horizontal lines);
2. a pool of cues (word phrases) displayed on a screen that may incidentally trigger spontaneous thoughts;
3. thought probes occurring at random times during which participants are asked to write down the content of their thoughts just before being stopped.

**Fig. 1:**
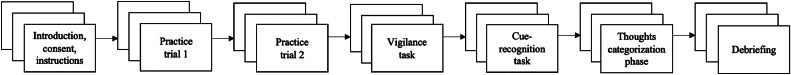
Schematic diagram of the computer procedure flow.

The computerized procedure, presented in this paper, encompasses elements beyond the vigilance task, and like the vigilance task, can be tailored to the requirements of a specific study. The detailed protocol employed in our study [28] is outlined in Fig. 1, which provides an overview of the general flow of the overall computer procedure.

### Basic information about the study (duration of the study, consents, etc.)

At the start of the program, participants enter their anonymity code and are provided with information about the general nature of the study. Key information is presented as follows:

- *The study explores cognitive mechanisms involved in sustained attention. It aims to investigate how attention fluctuates when individuals concentrate on a specific task for an extended period of time.*
- *If you agree to participate in this study, you will be asked to perform a computer task that involves: (a) responding to stimuli displayed on a monitor screen; (b) recording the content of your thoughts during this task. Additionally, you will be required to rate these thoughts on various scales.*

The participants are then provided with information about the privacy and confidentiality of the data, along with the consent form highlighting the option to withdraw from the study at any time. After reviewing this information, they can choose to participate in the study or opt out. If they consent, they proceed to complete a brief questionnaire asking for demographic information (e.g., date of birth, gender).

### Practice trial 1

Next, participants are informed that the study requires a high level of attentional focus. They are instructed to mute and hide their phones and to refrain from looking away from the monitor during the procedure. Subsequently, they are provided with the following instructions for the vigilance task:

- *You will see, on a computer screen, a large number of varying patterns of either horizontal or vertical lines. Your task is to respond to vertical lines only.*
- *Each time you see vertical lines on the screen, please press the red 'm' button on the keyboard.*
- *In addition to the lines, there will be some word phrases displayed in the center of the screen.*
- *The task you are participating in assesses the ability to concentrate on a monotonous task of detecting lines despite the simultaneous display of other stimuli, such as words and phrases.*

After receiving these instructions, participants engage in the first practice session, which includes 25 non-target stimuli (horizontal lines) and 2 target stimuli (vertical lines), without any thought probes. A detailed description of the layout of the displayed slides is available in the "Vigilance Task" section. If an individual does not achieve 50 % accuracy on the practice task, the training session is restarted. A comprehensive explanation of the method for calculating accuracy in this task will be presented in the section on data exclusion criteria. It is worth emphasizing that the use of two practice sessions allows us to progressively introduce participants to essential elements of the procedure, starting with instructions for the vigilance task and followed by guidance on how to report involuntary thoughts during the thought probes.

### Practice trial 2

Prior to the second practice trial, participants receive additional instructions. They are instructed to record the content of their thoughts when the program is stopped, regardless of the nature or perceived interest level of their thoughts.

- *As mentioned, this task evaluates individuals' ability to concentrate on relatively monotonous stimuli for an extended duration. Consequently, you may find yourself involuntarily thinking about other things during the task. This is entirely normal, given the mundane nature of the task. Information about the thoughts you may experience during the task is crucial for us, as it may enhance our understanding of people's ability to concentrate on monotonous stimuli.*
- *Therefore, while you are performing this task, the program will automatically stop at random intervals. Brief questions will then be displayed regarding your level of focus and the content of your thoughts at the particular moment when the program stops. Please be aware that these thoughts may pertain to various things – for example, simple associations, words, and facts.*

In studies of spontaneous thoughts, it is important to develop instructions that minimize the risk of accidentally priming participants' thoughts in a specific way. The instructions should avoid suggesting only the types of thoughts that researchers are investigating (e.g., spontaneous memories and future thoughts as was the case in the present study). At the same time, the instructions need to be comprehensive enough for participants to understand that they should report a broad spectrum of thoughts that does not narrow the scope of their thoughts in any way. To achieve this aim, the following additional instructions are provided:

- *They [thoughts] may also be more elaborate and may relate to current concerns, future goals, the current situation, or memories related to something from your personal past—something you have witnessed or experienced. There may also be thoughts that relate to the current situation of the study itself. All this content can be of very diverse nature, and some of these thoughts may relate to something concrete or specific. Other thoughts may describe something more general, abstract, or schematic. They may be thoughts that pop into your mind spontaneously, or they may be something you have deliberately chosen to think about. In this study, we are interested in thoughts of all types, regardless of their nature or content (i.e., what they are and what they are about).*
- *If the content of your thoughts is something you don't want to write about, then instead of describing that content, simply write an X and continue with the task. Remember, it doesn't matter if what pops into your mind is, in your opinion, interesting - just write it down.*

Participants then complete the second practice task, which is the same as the first one with the addition of one stop trial. When the program stops, participants are asked to provide a brief description of the content of their thought by typing it into the computer program. Additionally, they rate their level of concentration on a seven-point scale and specify whether the thought occurred deliberately (*they deliberately chose to think about it*) or involuntarily *(it simply popped into their mind spontaneously*). A comprehensive description of stop trials is provided in the "vigilance task" section. If a participant did not attain 50 % accuracy in the practice trial on the vigilance task, the practice trial is repeated. Following the completion of both training sessions, a brief summary of instructions is presented, and participants then advance to the main part of the study, namely, the vigilance task.

### Vigilance task

**Fig. 2:**
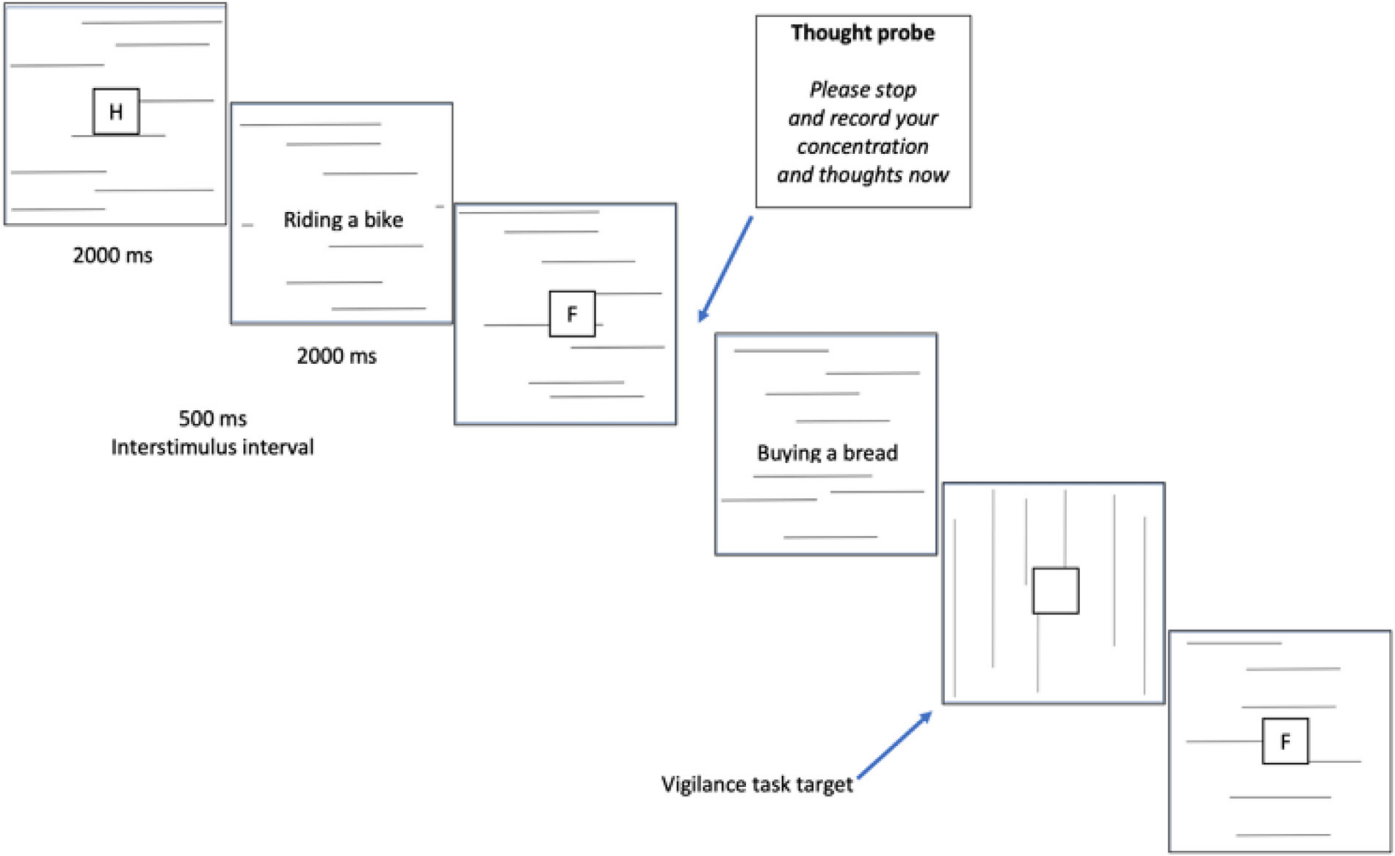
Vigilance task flow as used by Barzykowski, Ilczuk, & Kvavilashvili [28], slide by slide (the sequential cue presentation condition).

Consistent with a brief overview of the procedure, the vigilance task involves identifying patterns of vertical lines (15 target slides) within a sequence of 785 non-target slides with horizontal lines. A detailed depiction of the task flow in the vigilance task, slide by slide, is presented in Fig. 2. Each slide in the vigilance task is presented for 2 s, with short verbal phrases (e.g., *riding a bike*) presented only on some of the slides in center of the slide. The pool of 270 phrases^1^ comprises an equal number of neutral (e.g., *buying bread*), positive (e.g., *a wonderful smile*), and negative (e.g., *an unpleasant conversation*) phrases (90 phrases per each emotion category). The word-phrases are presented in two fixed pseudo-random orders and occur at varying intervals, consisting of a minimum of 1 (about 2.5 s) and a maximum of 7 (about 17.5 s) slides. The mean interval between the cues is about 3 slides (i.e., 7.5 s). Additionally, a square (approximately 1.5 cm by 1.5 cm) containing a random consonant (B, C, D, F, G, H, K, M, N, P, R, S, T, W, Z) is presented in the center of the screen on each trial with no word-cue. This element of the procedure was part of the parallel N-back task performed by the experimental groups and is not an essential component of the ongoing vigilance task used in the standard method (e.g., [23]).

The program stops automatically at 23 fixed points during the presentation. The probes, modelled on previous literature (Barzykowski et al. [[23], [24], [25], [27]]), are presented in a fixed pseudo-random order and occur at intervals of between 32 (about 80 s) and 40 (about 100 s) slides (the mean is 86.85 s). These intervals between the stops are comparable to similar previous studies (e.g., [29]; Barzykowski et al. [[23], [24]]).

Depending on the type and frequency of the thought being studied, the appropriate number of stops should be applied. During the stop, the following message appears on the screen:

- *Write down briefly, in a few words, what is on your mind. It does not matter at all whether you find it interesting or not. Everything is important regardless of what it is about. If, for some reason, you do not want to describe the content of your thoughts - write an X or describe them more generally. Use this, however, only on very rare occasions.*

Participants are asked to write down their thoughts briefly, in a few words, to prevent some participants from extending the procedure for an extended period (e.g., due to lengthy descriptions). This approach is also intended to minimise the risk of interfering with the natural flow and process of eliciting and reporting memories and thoughts. Once stopped, participants type a brief description of the content of their thoughts. They also rate how much they were concentrating on the task when stopped on a scale from 1 (*not at all*) to 7 (*fully concentrating*). The scale points are clearly labeled during the task. Additionally, participants specify whether the thought occurred deliberately (they decided to think about it) or involuntarily (it simply popped into their mind). After selecting either "*involuntarily*" or "*deliberately*," participants also specify what triggered the content by choosing from options such as 1=*something in the program*, 2=*something in my mind*, 3=*something in the surroundings*, 4=*nothing*. Furthermore, participants are asked to provide a brief description of the trigger. Throughout the experiment, additional attention control questions are used by asking participants to press specific number keys on the computer keyboard (e.g., when participants rate their concentration on the vigilance task in the thought probe, they may be presented with an additional question prompting them to press "3″ as an answer). This measure serves to identify inattentive participants, who could potentially be excluded from further data analyses (though, in our study, we did not exclude participants on that basis).

### Cue-recognition task

In our study [28], the unexpected cue recognition task was administered to participants immediately at the end of the vigilance task, with the instruction as follows:

- *In a moment, a word phrase will be displayed on the computer screen. Your task is to determine whether the phrase was presented on the computer screen while you were engaged in the concentration task.*

The cue-recognition task is a valuable but non-essential component of the procedure. In our study, it was employed to examine additional hypotheses concerning the connection between working memory load and the degree to which cues were noticed and processed. However, additional questions about cues presented during the vigilance task (which, may inadvertently trigger involuntary past and future thoughts during the vigilance task) may serve as an important measure to confirm whether the cues were noticed by participants.

In the cue-recognition task of our study, participants were exposed to a total of 84 cues, with half (42 cues) randomly selected from the pool of cues presented during the vigilance task. The remaining 42 cues were presented for the first time and were randomly selected from the remaining pool of 800 cues (for details, see Footnote 1) used in previous studies on spontaneous past and future thoughts (e.g., [[16], [26], [29], [30], [31]]). Importantly, each set of old and new cue phrases contained an equal number of neutral (*n* = 14), positive (*n* = 14), and negative (*n* = 14) phrases. The presentation of cues followed two fixed pseudo-random orders and was counterbalanced across groups and conditions. Participants responded to each cue displayed on the screen (*Yes* or *No*) at their own pace, without any time restrictions.

To assess the comparability of cues employed in the cue-recognition task, we computed the mean ratings for each phrase based on cue-phrase type (old phrases presented during the vigilance task and new phrases not seen before). Old and new cue-phrases did not significant differ from each other on ratings of concreteness, imagery, and typicality as shown by the results of three separate *t*-tests with cue-phrase type as an independent variable (all p_s_ > 0.53).

### Control questions

Following the cue-phrase recognition task, participants also answered additional manipulation-check questions. Initially, they provided responses to open-ended questions regarding their perception of the true goal of the study. This is a crucial step, as awareness that the study is centered on spontaneous memories and thoughts about the future could potentially lead to the intentional recall of such content. In addition, participants evaluated the perceived level of task-related fatigue, attention demand, task difficulty, and interest on 7-point rating scales. They also rated their overall concentration during the task, as well as their concentration specifically on verbal phrases, vertical lines, and the square with letters. Additionally, participants rated the perceived importance of performing the computer task to the best of their ability. Furthermore, participants provided ratings on the extent to which verbal phrases, and square-letters were experienced as interfering, along with how much they suppressed involuntary thoughts and ignored verbal phrases.

### Subsequent thought elaboration, categorization and rating phase

When first recording their thoughts, participants briefly describe their thoughts and do not categorise them as relating to their personal past (IAMs) or personal future (IFTs). Thus, it is essential to provide participants with an opportunity to further extend their initial thought descriptions and categorize/rate their recorded thoughts after completing the vigilance task, especially in the context of a subsequent coding process performed by expert judges. This ‘thought categorisation’ stage (see Fig. 1) allows participants to provide additional, more specific information about each recorded thought, contributing to the overall quality and the richness of the data. During this phase, participants review all the thoughts recorded during the vigilance task, one at a time, and in the same order as initially recorded (although some studies may present them in a random order, as in [6]). Simultaneously, they receive concise instructions outlining the nature of autobiographical memory and future thoughts, displayed on a computer screen alongside each thought. Participants are instructed to categorize each thought as an autobiographical memory, future-oriented thought, a thought related to the current situation, or another type of thought by clicking the appropriately labelled button. Once they decide, participants provide a longer, more detailed description of the thought and respond to several questions concerning various phenomenological characteristics (e.g., emotional valence, intensity of emotions, perceived importance) of each recorded thought by rating these characteristics on a 7-point scale (1 = low to 7 = high). The following instructions are provided:

- *Below is the content you typed in the first part of the study. If it is a memory, relating to something you have experienced, witnessed, or personally undergone, click "autobiographical memory". If it pertains to something from your future, such as plans or wishes (e.g., something you want or need to do) or events yet to happen, click "thought concerning the future". If the content is about the current situation, for example, "how much longer to go", click "current situation." If the thought is of a different nature and is not connected to your personal past, present, or future, click "other type of thought". There are no right or wrong answers here. Answer honestly and according to your own conviction. All subsequent questions apply only to the content written above.*

It is important to note that this step (i.e. a longer and more detailed description of the thought recorded during the initial thought probes) is provided as an additional step in our procedure after the vigilance task is completed. Although we consider this step essential for improving the quality of data coding, it is not mandatory, as many other studies employing similar laboratory methods have not included it in their procedure (e.g., [[9], [23]]). However, it can be a valuable tool in studies specifically focusing on the contents of memories, as demonstrated by Barzykowski et al. [27,32].

After the categorization phase, participants are provided with detailed information about the study they participated in, and are thanked for their involvement.

## Customizability of the procedure

Above, we concentrated on the basic version of the vigilance task. However, it is important to emphasize that the basic experimental procedure can be modified in various ways and adapted to specific goals of a particular study investigating involuntary thoughts. There are several possibilities for modifying the procedure. For example, a researcher may be interested in manipulating cue features, such as the number of positive and negative cues or the frequency of cue presentation. The program can also be expanded to include accompanying concurrent tasks. An example of such a modification could be the addition of a task that manipulates the working memory load. This variant of the task was utilized in the experimental groups in our study (for other manipulations, see [33,34]). Specifically, during the vigilance task, a square (approximately 1.5 cm by 1.5 cm) containing a random consonant (B, C, D, F, G, H, K, M, N, P, R, S, T, W, Z) was presented in the center of the screen on each trial with no word cue. Participants were instructed to respond by pressing a green button (“X” on the keyboard) each time the letter currently presented on the screen was the same as the one presented either 1 trial ago (low working memory load condition) or 3 trials ago (high working memory load condition). In the modified procedure, participants were exposed to a series of consonants (in uppercase: B, C, D, F, G, H, K, M, N, P, R, S, T, W, Z), one by one, presented in a square appearing in the center of the screen for 2 s with an interstimulus interval of 0.5 s. Various further modifications of the vigilance task are possible depending on the objectives of the given study.

## Working with data

**Fig. 3:**
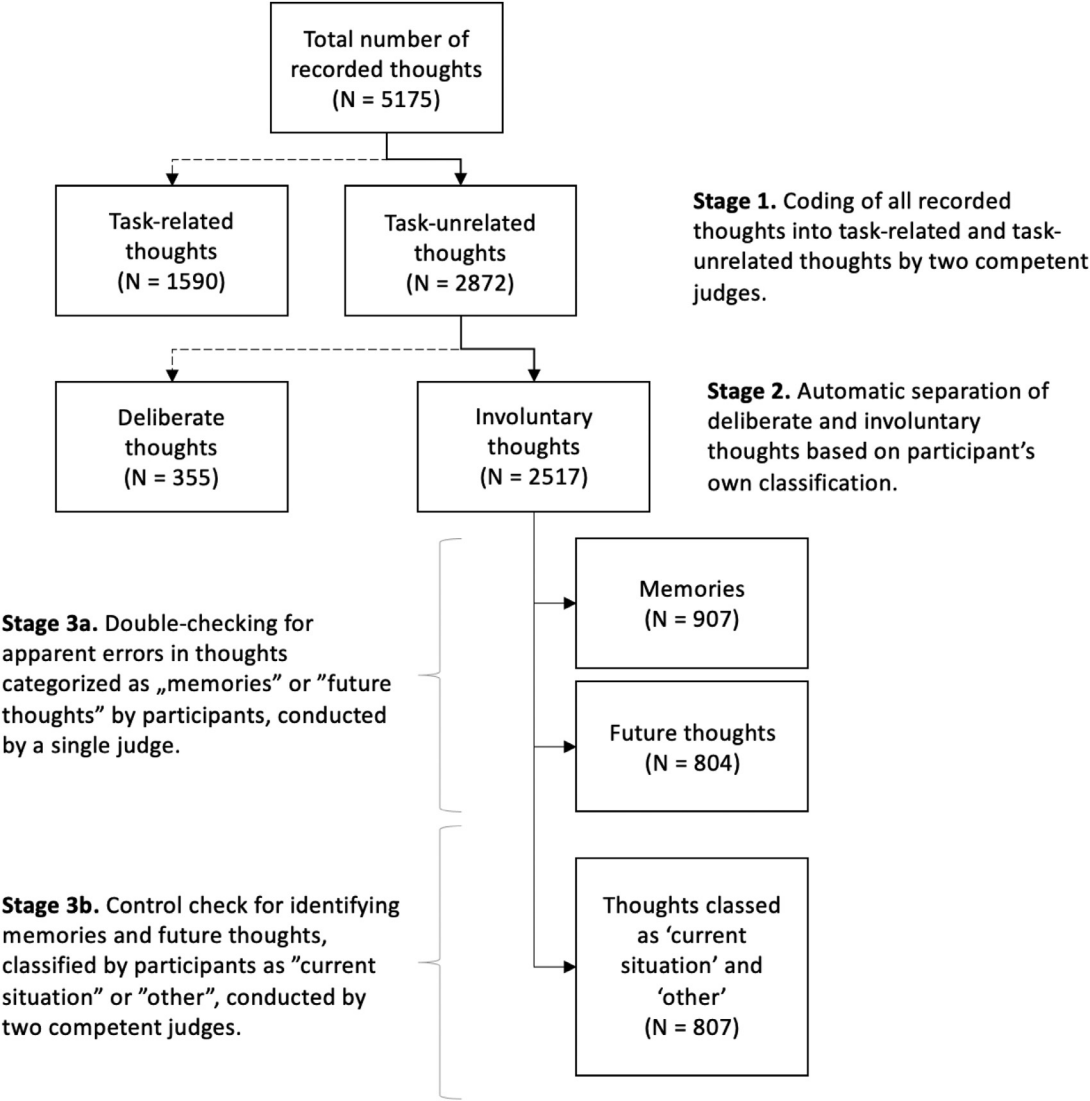
Step-by-step categorization and coding of the data at each stage.

In the Supplementary Material, we provide a comprehensive workbook that includes examples of data and a step-by-step coding process. The workbook is organized into six separate sheets: (1) S1_Raw data: This sheet contains the original, unprocessed data; (2) S2_TUT vs TRT: This sheet presents participants' entries coded as task-unrelated and task-related thoughts; (3) S3_deliberate vs. involuntary (thoughts): This sheet presents participants’ own classification of recorded thoughts into thoughts that they intentionally chose to think about and those that popped into their mind spontaneously (i.e., without a prior intention to think about them); (4) S4_other vs. current situation: This sheet categorizes data into thoughts about the future and past versus thoughts about other situations and the current situation. This classification facilitates further processing in steps 5 and 6; (5) S5_IAMs & IFTs (control check): This sheet implements a control-check procedure for thoughts categorized as 'current situation' or 'other,' aiming to identify thoughts about the past or future within these categories; (6) S6_IAMs & IFTs (double check): This sheet employs a double-check procedure for thoughts categorized as 'past' or 'future' and the purpose of this double-check is to scrutinize respondents' categories and identify any potential errors. In addition, Fig. 3 presents a schematic view of the data workflow.

### Exclusion criteria

To increase the validity of the data, several exclusion criteria can be applied. In the present study, we excluded participants if a technical problem during the experimental session prevented them from successfully completing the vigilance task. Data of participants who scored less than 50 % correct in the vigilance task and/or the N-back task, indicating a lack of engagement in task performance, were also excluded from the analyses. The performance on the vigilance task was calculated by dividing the number of correctly detected targets (slides with vertical lines) by the total number of target stimuli. In addition, when analyzing reaction times (RTs) to targets in the vigilance and the N-back tasks, RTs under 100 ms were excluded, due to a threshold of 100 ms being considered as the minimum for valid and genuine responses (e.g., [35]). Finally, participants who correctly guessed the true purpose of the study (i.e., the main interest in past and future thoughts) were excluded from the analysis concerning the frequency of involuntary task-unrelated thoughts, including IFTs and IAMs. Based on these criteria, out of 240 participants, only 15 participants (approximately 6 %) were excluded from the study: one participant due to technical problems, 11 participants because they had less than 50 % correct responses in the vigilance or N-back tasks, and 3 who had guessed the true purpose of the study.

### Categorization process

The key data obtained during the study consist of thoughts reported by participants and their characteristics (see Table 1 below and the S1_Raw data sheet in the Supplementary file for a detailed breakdown of the raw data). These include:

- *A short description of the thought, provided by participants when being stropped during the vigilance task.*
- *Information on whether the thought was involuntary or deliberate.*
- *Information on what triggered the thought.*
- *A longer (more detailed) description of the thought, clarifying the thought content, recorded during the initial thought probes.*
- *Information on the temporal focus of the thought provided by the participant (autobiographical memory, future-oriented thought, thought about the current situation, other).*

**Table 1:** Examples of thought data and categorisations provided by participants. See also workbook.xlsx file (supplementary materials no 1).

Participant ID	Thought ID	thought	trigger	Long description	Deliberately vs involuntary	Temporal category
1	1	*I'm waiting for the vertical lines*	*doing the task*	*I was waiting for the vertical lines because they appeared very rarely*	deliberately	present
1	2	*My boyfriend*	*phrase on the screen*	*My dear boyfriend, I saw with last night*	involuntary	past
1	3	*X*	*my life*	*X*	involuntary	past
2	4	*So many lines*	*task*	*I was weary of the task and the fact that I kept seeing lines*	involuntary	present
2	5	*My favourite song in my head*	*I don't know*	*I was humming a Rediohead band song in my head*	involuntary	other
2	6	*University*	*Phrase "school"*	*I would like to pass the upcoming exams*	involuntary	future

As part of our study (with a final sample of 225 participants), we collected a total of 5175 thoughts. The data were sorted in alphabetical order based on the first letter of the memory description and subsequently processed through the following stages: (1) distinguishing task-related and task-unrelated thoughts, (2) distinguishing involuntary and voluntary task-unrelated thoughts, and (3) distinguishing memories and thoughts about the future. Each stage is illustrated in Fig. 3 and will be described in detail below.

#### The first coding stage: distinguishing between Task-Unrelated Thoughts and Task-Related Thoughts

The initial step in processing the data involved segregating participants' thoughts related to the ongoing vigilance task from those unrelated to the task. Two competent judges coded the data. Both judges received extensive training to ensure a solid understanding and proficiency in coding. To achieve this, they: (1) were provided with coding definitions (see below), which were discussed, to ensure a good understanding of the categories, and any doubts were resolved prior to the data coding phase; (2) were asked to code a small number of entries, followed by a discussion about the categories, ensuring any misunderstandings and/or doubts about the coding system were addressed through discussion; (3) were asked to code all remaining entries once they declared full understanding of how to use the codes; and (4) were asked to discuss any disagreements to arrive at final agreed-upon categories for thought descriptions.

To ensure the integrity and reliability of the coding process, the judges were chosen based on their expertise in cognitive psychology and the basic knowledge of coding methodologies. Prior to their involvement in the study, the judges underwent thorough instruction regarding the coding scheme and methodology. This training session aimed to familiarize them with the coding criteria and ensure a consistent understanding of the task at hand. In addition, to minimize potential biases and ensure impartiality in the coding process, the judges were blind to the study hypotheses and specific data being analyzed. Overall, the selection and training of judges, and the blind coding procedure were integral components of our methodological approach, designed to enhance the accuracy and reliability of the data.

It is important that a longer description of each thought serves as an aid in interpreting the thought that was recorded during the vigilance task and it cannot be relied on exclusively when determining the final coding category for the thought. For example, consider a situation in which a person, during the vigilance task, noted the following thought: *'I wondered if I would get the job at the company*'. In a longer description, they further elaborated: *'I was curious if I would get a job at the company where I had an interview a week ago*'. The example provided was categorized as a 'task-unrelated thought' and a 'future thought'. This is because the thought typed during the vigilance task explicitly refers to the future (i.e., getting a job), and only later does the participant expand their description to include additional threads from the past. In such a situation, the thought entered during the vigilance task should be considered decisive.

The coding task involves categorising thoughts into one of the following categories:

- *Task-Related Thoughts (TRTs): These are defined as thoughts that are clearly related to the ongoing task being performed, being fully in 'here and now', and therefore, are directly related to the task at hand (e.g., I'm waiting for the vertical lines, I pressed the red button).*
- *Task-Related Interference (TRIs): Thoughts in this category involve an appraisal of the task or one's performance on the task. Examples of TRI include any references to aspects of the vigilance task (e.g., I was wondering how many different patterns of lines are being used in this experiment; remember to press the space bar, but there are not many vertical lines), any mention of the phrases on the screen (e.g., swimming in the pool was spelled wrong), or any reference to a state/emotion that arose in response to the vigilance task (e.g., I'm feeling quite anxious about the words).*
- *Task-Unrelated Thoughts (TUTs): These involve thoughts with no relationship to the task at hand or the current situation. These are thoughts that do not contain any explicit reference to the vigilance task and may pertain to the past (e.g., memory of attending a music festival with friends at the age of 19; romantic dinner at a cozy restaurant by the beach with my boyfriend last summer), present (e.g., thinking about my parents' current journey to visit me, feeling cold in the room), or future (e.g., need to start my project after the Christmas break; upcoming exam I have in a few weeks during the examination session).*

The judges were initially tasked with coding the first 200 thoughts to verify their understanding of the instructions before coding all the remaining thoughts. If their agreement, measured by the percentage of thoughts assigned similar codes, was higher than 80 %, they were asked to proceed and code the remaining thoughts. If it was lower, they received clarifying instructions, primarily focusing on those categories they consistently assigned differently.

To ensure the consistency of judgments made by the independent judges involved in data coding, the inter-rater reliability was assessed by calculating the Cohen's Kappa coefficient. Despite some variability in views about what counts as a good level of agreement between judges when using Cohen's Kappa, Landis and Koch [36] have suggested the coefficients of 0.60 to 0.80 to be indicative of substantial agreement and of 0.81 to 1.00 – perfect agreement. Based on this recommendation, in our study, judges achieved an excellent inter-rater reliability at this final coding stage (Cohen's Kappa = 0.84), suggesting robust consistency in the coding process. Any discrepancies or disagreements between judges were resolved through discussion and consensus to ensure the accuracy and reliability of the final data. As a result of this coding, we obtained 2872 Task-Unrelated Thoughts (TUTs) and 1590 Task-Related Thoughts (TRTs) and Task-related Interference (TRIs). An example of this part is presented in Sheet S2 in the supplementary file.

#### Distinguishing between deliberate and involuntary task-unrelated thoughts

Among the task-unrelated thoughts, all thoughts that respondents classified as involuntary were included into further analyses. This stage was automated, relying on the participants' classifications (see Sheet S3 in the supplementary file). As a result, we obtained 2517 involuntary task-unrelated thoughts, which were then submitted to competent judges for the second stage of coding.

#### The second coding stage: distinguishing between involuntary autobiographical memories (IAMs), involuntary future thoughts (IFTs) and other thoughts

The aim of the final stage was to identify reported thoughts as IAMs and IFTs with as much precision as possible. As described earlier, and in line with previous studies (e.g., [23]), it was participants themselves who provided information about the temporal focus of their recorded thoughts by indicating if their thought referred to past, future, present or any other type of event. Thoughts categorised by participants as IAMs or IFTs and those classified as ‘present’ or ‘other’ were subjected to two separate data coding procedures, a double-check and a control-check procedure, respectively.

A double-check procedure (Sheet S6 in the Supplementary file) was employed only for thoughts categorized by participants as either IAMs or IFTs. A competent judge screened all thoughts in the two categories, aware of the category to which the thought was assigned by the subject. As highlighted by Barzykowski & Niedźwieńska ([33], p. 123; also Barzykowski et al. [27], p. 676), due to the computerized nature of the vigilance task, the decision regarding which category the mental content belonged to is irreversible, which can lead to some mistakes made by participants when categorizing thought descriptions. Therefore, in this part of coding procedure, the judge's task was to check the respondents' categories for any obvious mistakes. In cases of doubt, the final category was determined by the participant's own classification. As a result, 20 thought categorisations were changed. Some were described by participants themselves as being accidentally misclassified by pressing the wrong button, while for others, the description left no doubt that the participant had made an error when, for example, the thought ‘*seen yesterday's episode of the series with my boyfriend*’ was classified as a thought of the future. Other examples with descriptions can be found in Supplementary File S6.

A different approach, namely, a control check procedure, was taken for the thoughts classified by participants as ‘other’ and ‘current situation’ (see sheet S5 in the Supplementary file). Examination of participants thought descriptions shows that they often choose the 'other' category when experiencing atemporal thoughts (e.g., *does dwarfs exists?*) or when uncertain about how to classify their thoughts. This uncertainty may arise from the thoughts encompassing events from different time frames (e.g., *I thought that I have just pressed correctly the button and also I thought of the lunch I will eat after the study is over*). Similarly, participants may select the 'current situation' category even when a thought has minimal relevance to the present moment (e.g., *I'm sad because I was reminded of yesterday's conflict*). As a result, some of the past and future thoughts end up mis-classified by participants into these two categories. From the researcher's perspective, the mere appearance of a thought potentially involving the category of interest (i.e., IAMs or IFTs) is significant, regardless of whether it appears in the company of thoughts of a different temporal nature. For this reason, a control-check procedure was chosen, in which two trained judges re-coded the thought descriptions that were classed by participants as ‘other’ or ‘current situation’. Importantly, the judges were blind to the category assigned by participants. They were instructed to classify thoughts into the following categories:

- *Memories: Something concerning the past, an event that happened, took place, or was experienced or witnessed.*
- *Future thoughts: Something regarding the future, including wishes, plans, something to do (e.g., something one wants or needs to do), or events that are yet to take place,*
- *Other: Thoughts that do not fall into the categories of memories, or future thoughts.*

They were also instructed to always assign the category 'other' when in doubt. The judges were initially tasked with categorizing the first 200 thoughts to verify their understanding of the instructions before categorizing all thoughts. If their agreement was higher than 80 %, they were asked to code the remaining thoughts. If it was lower, they received clarifying instructions, primarily focusing on those categories they consistently assigned differently. Inter-rater reliability, as measured by Cohen's Kappa, for this portion of data coding was acceptable (*κ* = 0.66). As a result of this coding, there were 907 thoughts coded as IAMs, 804 coded as IFTs, and 807 thoughts classified as 'other' than IAMs and IFTs. The numbers of Involuntary Task-Related Thoughts (task oriented + task-interference thoughts), IAMs, and IFTs were then used in statistical analyses to verify the research hypotheses.

Having a number of IAMs and IFTs for each participant allowed us to further analyse the frequency of such spontaneous thoughts between groups and experimental manipulations of working memory load. For instance, in Study 1 by Barzykowski et al. [28], we examined the effects of the working memory load and cue-presentation on the number of IAMs and IFTs reported during the vigilance task. Working memory load involved either a 1-back or 3-back task with letters presented in the square in the centre of the slides (see Fig. 2) and the control condition in which participants performed only the vigilance task (detecting slides with vertical lines) without the additional N-back task. Cue presentation was manipulated by presenting incidental cue-phrases on slides that did not contain a square with a letter inside (as depicted in Fig. 2) or presenting them together with the N-back task stimuli (sequential versus simultaneous cue presentation conditions, respectively). The numbers of IAMs and IFTs were separately subjected to two-way ANOVAs with working memory load (no load, 1-back, 3-back) and cue-presentation (simultaneous, sequential) as between subjects variables. Significant main effects of working memory load were observed for both IAMs (*F* (2, 214) = 3.84, *η2* = 0.03, *p* = .023) and IFTs (*F*(2, 214) = 7.73, *η2* = 0.07, *p* = .001). Post hoc analyses for IAMs showed that participants in the control condition reported significantly higher number of IAMs than those in the 1-back and 3-back task conditions (*p_s_* < 0.046) who did not differ from each other (*p* = .615). For IFTs, their frequency was significantly higher in both the control and 1-back working memory conditions compared to the 3-back condition (*p_s_* < 0.001), but the control and 1-back condition did not differ from each other (*p* = .956). No other main or interaction effects were significant for IAMs and IFTs.

## Conclusions

In this paper, we have outlined a protocol for a comprehensive procedure to collect and coding involuntary past and future-oriented thoughts. The protocol describes two main stages in the procedure: data acquisition and data processing. In the data collection phase, we presented an easily modifiable computerized version of the vigilance task, designed for broad application in various studies that focus on eliciting and measuring involuntary thoughts in controlled laboratory conditions. For data preparation and coding, we have described a detailed procedure for categorizing and coding different types of thoughts, involving the use of participants and competent judges. Additionally, we have highlighted some of the difficulties that may be encountered in this categorization/coding process.

## Declaration of generative AI and AI-assisted technologies in the writing process

During the preparation of this work, Krystian Barzykowski used ChatGPT 3.5 to proofread sentences and enhance the language and readability. After using this tool, the author reviewed and edited the content as needed and takes full responsibility for the content of the publication.

## Ethics statements

The Research Ethics Committee approved the Study. Written consent for participation was obtained prior to data collection. Participants were informed that they were free to withdraw from the study at any point.

## CRediT authorship contribution statement

**Krystian Barzykowski:** Conceptualization, Methodology, Validation, Formal analysis, Resources, Data curation, Writing – original draft, Visualization, Supervision, Funding acquisition. **Ewa Ilczuk:** Conceptualization, Methodology, Resources, Investigation, Writing – original draft, Visualization, Project administration. **Lia Kvavilashvili:** Conceptualization, Methodology, Writing – review & editing.

## Declaration of competing interest

The authors declare that they have no known competing financial interests or personal relationships that could have appeared to influence the work reported in this paper.

## Data Availability

No data was used for the research described in the article.

## Appendix Supplementary materials

Image, application 1

Supplementary material and/or additional information Image, application 2

## Footnotes

1 The pool of phrases, used in the study, was created from the word-phrases originally used in the 17 study. As described elsewhere (16), the 800 cue-phrases were translated into Polish by three independent research assistants proficient in English. In addition, a Polish linguist thoroughly reviewed the translations for grammar, inflection, and phraseology. All phrases were evaluated for neutrality, positivity, or negativity by twelve independent coders. Following the methodology of 17, we employed the percentage of agreement on perceived phrase type as a measure of inter-coder reliability. Phrases with an inter-coder agreement of 66 % or more (indicating that a verbal phrase was classified as neutral if at least 8 out of 12 coders rated it as neutral) were included in the final pool of 800 phrases. This pool consisted of roughly equal numbers of neutral (N = 267), positive (N = 267) and negative (N = 266) phrases.
